# Effect of Long-Term Storage Temperature on the Quality of Extra-Virgin Olive Oil (Coratina cv.): A Multivariate Discriminant Approach

**DOI:** 10.3390/antiox14111379

**Published:** 2025-11-19

**Authors:** Pasquale Crupi, Maria Lisa Clodoveo, Addolorata Desantis, Roberta Zupo, Filomena Corbo

**Affiliations:** 1 Department of Agricultural, Food and Forestry Sciences, University of Palermo, Viale delle Scienze, 13, 90128 Palermo, Italy; pasquale.crupi@unipa.it; 2 Interdisciplinary Department of Medicine, University of Bari Aldo Moro, Piazza Giulio Cesare, 11, 70124 Bari, Italy; zuporoberta@gmail.com; 3 AGRIDÈ S.r.l., Applied Research in Agri-Food Technologies, Via G. Matteotti, 108, Bitonto, 70032 Bari, Italy; addolorata.desantis@uniba.it; 4 Department of Pharmacy–Drug Sciences, University of Bari, E. Quagliarello University Campus, Via Orabona, 4, 70125 Bari, Italy; filomena.corbo@uniba.it

**Keywords:** extra virgin olive oil, Coratina cultivar, storage temperature, oxidative stability, polyphenols, kinetic modeling, multivariate analysis

## Abstract

Kinetic evolution of quality parameters in 21 extra-virgin olive oils (EVOOs) from Coratina cultivar was evaluated during 18 months of dark storage at room temperature (RT) and 4 °C (LT). The aim was to identify the most discriminating variables—fatty acids, peroxide value, spectrophotometric indices, and phenolic compounds—using kinetic analysis and multivariate statistics. Fatty acids remained stable, while peroxide value and ultraviolet absorbance indices increased significantly at RT, following zero-order kinetics. Polyphenols declined markedly after 6 months, especially at RT, with degradation rates influenced by initial concentrations. Hydroxytyrosol and tyrosol followed pseudo-zero-order kinetics, whereas secoiridoids and lignans followed second-order kinetics. Discriminant analysis achieved 90% accuracy (*p* = 0.000012) in classifying oils by storage condition. The most relevant discriminants were associated with phenolic degradation and oxidative changes. These findings support the importance of low-temperature storage in preserving the biochemical quality and shelf life of EVOOs.

## 1. Introduction

Extra-virgin olive oil (EVOO) traditionally represents one of the cornerstones of healthy dietary habits, such as in the Mediterranean diet. This is mainly due to the presence of high levels of monounsaturated fatty acids (mostly oleic acid) in the saponified fraction as well as bioactive compounds as minor components, such as polyphenols, associated with well-demonstrated health-promoting capacity [1,2,3,4]. Several factors may influence the quali–quantitative composition of EVOOs, including cultivar and harvesting time and also processing technologies and storage conditions [5,6].

According to Annex I of Delegated Regulation (EU) 2022/2104 (http://data.europa.eu/eli/reg_del/2022/2104/oj, accessed on 21 July 2025) and International Olive Oil Council (IOC) trade standard, olive oil must comply with some quality indices—free acidity (FA), peroxide value (PV), K_270_, K_232_, ∆K in spectrophotometry, etc.—to be classified in the extra-virgin category; however, this compliance can be seriously compromised because of the degradation reactions, occurring during storage of EVOO, which might provoke the downgrade of the product [7,8]. The high oleic acid content and abundance of natural antioxidants, particularly polyphenols, in EVOO contribute to its oxidative stability by scavenging free radicals and mitigating oxidative processes [9,10]. However, depending on the storage conditions, such as inadequate temperature, light, or oxygen, the quality of EVOO may vary, and the phenolic content can be altered or diminished by a series of chemical reactions, which threaten the stability of the olive oil [11]. Many researchers have demonstrated that the degradation rate of phenolic compounds during oil aging is strongly dependent on the storage temperature and their initial concentration [12,13]. Storage of EVOOs at room temperature led to an increase in simple phenols over three months [14]. After 18 months, a substantial loss of secoiridoids occurred; this decrease was nearly 50% in oils with low initial concentrations of minor polar compounds, compared to only 20% in those with high initial concentrations [15]. Furthermore, in a long storage of low, medium, and high-phenolic EVOOs under different temperatures, cold storage (4 °C) effectively retarded the oxidation and hydrolysis of secoiridoids, an effect that persisted as long as oxygen availability remained limited [16,17].

Several studies on oxidative stability and the evolution of phenolic compounds during storage have utilized the Coratina olive cultivar, which is recognized for its high polyphenol biosynthetic capacity [18]. Of particular interest is the work of Macciola and De Leonardis (2022) in Molise, which confirmed the exceptional oxidative stability and long-term food durability of Coratina oil [19].

In recent years, multivariate strategies based on unsupervised methods, such as principal component analysis (PCA) and multivariate analysis of variance (MANOVA), as well as multivariate pattern recognition methods like linear discriminant analysis (LDA), have been developed to allow for a more complete interpretation of data structures, particularly in determining the shelf life and stability of EVOO, whether under ambient or forced conditions. In these targeted analytical approaches, multiple related analytical parameters, rather than a single parameter or measurement, of a specific food quality-related reaction are evaluated as a function of the extrinsic process variables [20].

Therefore, starting from the hypothesis that lower storage temperature should slow the depletion kinetics and degradation rates of both commodity and quality parameters in EVOO, the main objective of this stability study was to identify the principal variables (among FA, PV, K_232_, K_270_, ΔK, simple phenols, lignans, and secoiridoids) responsible for a reliable discrimination of 21 samples of Coratina *cv.* EVOOs (from three consecutive seasons), stored at room temperature (RT) and 4 °C (LT) for 18 months, which is still commonly reported as the “best before” date of minimum durability, by using a multivariate (MANOVA, PCA, and LDA) discriminant approach. We chose to sample EVO oils from Coratina *cv.* because they are the most important and widespread variety in Puglia and, especially, the most present oils, both in terms of frequency of lots and volume, available on the Italian market. In this context, the novelty of this research lies in the integration of kinetic analysis with multivariate statistics to move beyond descriptive observation to a mechanistic and predictive understanding of olive oil degradation. This can provide a powerful, evidence-based framework for the olive oil industry to optimize storage conditions, validate shelf life, and protect the quality and value of a premium product like Coratina EVOO.

## 2. Materials and Methods

### 2.1. Chemicals

Methanol, acetonitrile, and water (HPLC grade) were purchased from Chromasolv—VWR International Srl (Milano, Italy); ortho-phosphoric acid 85%, diethyl ether, absolute ethanol, phenolphthalein, sodium hydroxide, potassium iodide, chloroform, acetic acid (glacial), starch paste, sodium thiosulphate, Folin–Ciocalteu reagent, and iso-octane were purchased from CARLO ERBA Reagents Srl (Cornaredo-Milano, Italy). Tyrosol and syringic acid were purchased from Merk KGaA (Darmstadt, Germany).

### 2.2. Extra-Virgin Olive Oil Sample Collection

Twenty-one samples of EVOO (Coratina *cv.*) analyzed in this study were provided from different producers during three consecutive seasons: 2020 (3COL, CICO, P18, C33, D02, and D03), 2021 (6C, 11C, 12C, 13C, 14C, and 16C), and 2022 (1C/22, 2C/22, 3C/22, 4C/22, 5C/22, 7C/22, 8C/22, 10C/22, and 16C/22). EVO oils were produced between mid-October and mid-November (for each season) with green olives. The milling process occurred with two-phase technological systems of different brands using knife and hammer crushers. EVO oils were sampled after milling and filtration so that their aging time could initially be considered zero (t_0_).

### 2.3. Storage Conditions of EVOO Samples

The samples collected each year were divided into aliquots packaged in 100 mL dark glass bottles with screw caps, labeled with the assigned sample code and divided into two storage methods: (1) at room temperature (RT) and, as indicated by the legislation, “in cool place away from sources of light and heat” at controlled temperature of 25 °C; (2) stored in a refrigerator at 4 °C (LT), completely in the dark. The temperature of the refrigerator was checked daily with a calibrated thermometer. Then, each sample was chemically analyzed at t_0_ and, consecutively, every 6 (t_1_), 12 (t_2_), and 18 (t_3_) months.

### 2.4. Qualitative Indices Determination

Free acidity, peroxide value, K_232_, K_270_, and ΔK were determined using the analytical methods described in EU Reg. 2105/2022 (http://data.europa.eu/eli/reg_del/2022/2105/oj, accessed on 21 July 2025). Spectrophotometric analyses for measuring absorbances at 232, 266, 270, and 274 nm of 0.25 g of olive oil diluted with iso-octane in a 25 mL volumetric flask were conducted by a Shimadzu UV 2600i spectrophotometer (Kyoto, Japan).

### 2.5. Extraction and HPLC-DAD Determination of Phenolic Compounds

The extraction of phenolic compounds from the selected EVOOs was performed by mixing 2 g of oil with 5 mL of methanol/water (80:20 *v*/*v*). The mixtures were then vortexed for 1 min, treated in an ultrasonic bath for 15 min at room temperature, and centrifuged at 5000 rpm for 25 min at 20 °C. The resulting hydroalcoholic phase was recovered and filtered using syringe PTFE filters (0.45 µm). Before the injection, 20 µL of internal standard (IS), syringic acid (0.015 mg/mL), was added to 1 mL of each extract. Separation and identification of polyphenols were carried out, according to the specificities reported in the method COI/ T.20/Doc No 29, 2009 (https://www.internationaloliveoil.org/wp-content/uploads/2022/10/COI-T20-Doc.-29-2009-Eng.pdf, accessed on 21 July 2025), by using an HPLC Infinity 1260 system equipped with a degasser, quaternary pump (G7104C), autosampler (G7129C), thermostatic column compartment (G7116A), and Diode Array Detector (G7115A) (Agilent Technologies, Palo Alto, CA, USA). EVOO extracts were injected onto a reversed stationary phase column, Spherisorb ODS-2 RP-C18 (250 × 4.6 mm i.d., particle size 5 μm, Waters, Sesto San Giovanni, Italy) protected by a C18 Guard Cartridge (4.0 × 2.0 mm i.d., Phenomenex, Torrance, CA, USA). Water 0.2% H_3_PO_4_ (*v*/*v*) (solvent A), methanol (solvent B), and acetonitrile (solvent C) were used as mobile phase following a ternary linear elution gradient: 0 min, 96% A—2% B—2% C; 40 min, 50% A—25% B—25% C; 45 min, 40% A—30%B—30% C; 60 min 0% A—50% B—50% C; 70 min, A—50% B—50% C; 72 min, 96% A—2% B—2% C; 82 min, 96% A—2% B—2% C. Flow rate and column temperature were set at 1 mL min^−1^ and 25 °C, respectively.

The content of 3-hydroxytyrosol (3,4-DHPEA), tyrosol (*p*-HPEA), oleacein (3,4-DHPEA-EDA), oleocanthal (*p*-HPEA-EDA), lignans (pinoresinol + acetoxypinoresinol), oleuropein aglycone (3,4-DHPEA-EA), and ligstroside aglycone (*p*-HPEA-EA) was determined according to the formula reported in the method COI/ T.20/Doc No 29, 2009 and expressed in mg/kg of tyrosol equivalents.

Total polyphenols (TPPs) were determined according to the method by Clodoveo et al., 2016 [21]: 100 µL of phenolic extract or standard were pipetted into a 10 mL test tube and mixed with 100 µL 2 N Folin–Ciocalteu reagent and, after 5 min, with 800 µL of Na_2_CO_3_ 5%. The mixture was held at 40 °C for 20 min and then at room temperature for 15 min. The total phenol content was determined colorimetrically at 750 nm using a Lambda Bio 20 UV/Vis Spectrometer Perkin Elmer (Milan, Italy). The results were expressed as mg of gallic acid per kg of oil. A calibration curve of gallic acid was acquired in the concentration range of 10–100 μg/mL (R^2^ = 0.978).

### 2.6. Kinetic Data Analysis

Data of qualitative indices and polyphenols determined at different storage time and temperature were elaborated by using zero-order (1), first-order (2), and second-order (3) reaction models and the rate constant values were computed from the following equations:

(1) $$I=I_{0}+k_{0}t$$

(2) $$lnI=lnI_{0}+k_{1}t$$

(3) $$\frac{1}{I}=\frac{1}{I_{0}}+k_{2}t$$

where I is the selected measure, k_0_, k_1_, and k_2_ are the zero-order, first-order, and second-order rate constants, t is the storage time in days, and I_0_, lnI_0_, and 1/I_0_ are the intercepts. The order of the reactions was evaluated by visual inspection (based on the determination coefficient, R^2^, closest to 1 and the residual analysis) of the plots of I, ln I, and 1/I against t.

### 2.7. Statistical Analysis

A first data matrix, composed of the qualitative indices and polyphenols values (as the average of duplicate measurements) registered in Coratina EVOOs at different storage conditions during the three years of study, was statistically analyzed by applying Shapiro–Wilk’s W test together with Levene test to testing their normal distribution and homoscedasticity and full factorial two-way MANOVA to determine the influence of the two factors, storage time, and temperature on the measured parameters (Table S1—Supplementary Information) and a principal component analysis (PCA) as applied to qualitative indices and polyphenols values recorded at t_0_ to clusterize the EVOOs from the three seasons. Pearson correlation analysis was carried out between the concentrations of 3,4-DHPEA and *p*-HPEA, and 3,4-DHPEA-EA and *p*-HPEAEA, respectively. Subsequently, a second data matrix, composed of k rate constants of the qualitative indices and polyphenol evolution during storage time at different temperatures, was statistically analyzed by performing a forward stepwise linear discriminant analysis (LDA) to assess which variable(s) are the best predictors between oils stored at RT and LT. The stepwise selection procedure was configured with a maximum number of steps equal to the total number of variables. The criterion for variable inclusion was a unique contribution to group discrimination, as measured by an F-statistic exceeding the user-defined entry threshold of 1. To mitigate multicollinearity, a tolerance value of 0.01 was set, ensuring that variables explaining more than 99% of the variance in another variable were excluded to preserve model stability. Significance was determined at *p* < 0.05. All statistical treatments were computed using the STATISTICA 12.0 software package (StatSoft Inc., Tulsa, OK, USA).

## 3. Results and Discussion

### 3.1. Evolution of Trade Quality Parameters of Coratina cv. EVOO During Storage

Conventional trade quality indices, including FA, PV, K_232_, K_270_, and ΔK measured in Coratina variety EVOO from three consecutive seasons (2020–2022) and stored at two different temperatures (RT and LT) for 6 (t_1_), 12 (t_2_), and 18 (t_3_) months, are reported in Table 1.

No significant influence of both storage temperature and storage time was observed for FA during the three years of study (Figure S1—Supplementary Information); the data indicated that, at both investigated temperatures, the hydrolytic activity of the residual lipase enzymes following extraction was comparable, a result which contrasts with the existing literature [11,22]. The values of FA were coherent with those previously reported in Coratina oil [17] and, in all conditions, they did not reach the maximum (0.8%) allowed by IOC (IOC/T.15/NC No 3/Rev. 20 November 2024) for EVOO category; in particular, samples from the 2022 season exhibited higher percentage of FA compared to the more similar 2020 and 2021 samples. This was likely attributable to climatic conditions, specifically elevated humidity and precipitation immediately prior to the olive harvest, which promoted fungal diseases and olive fly infestations.

**Figure 1:**
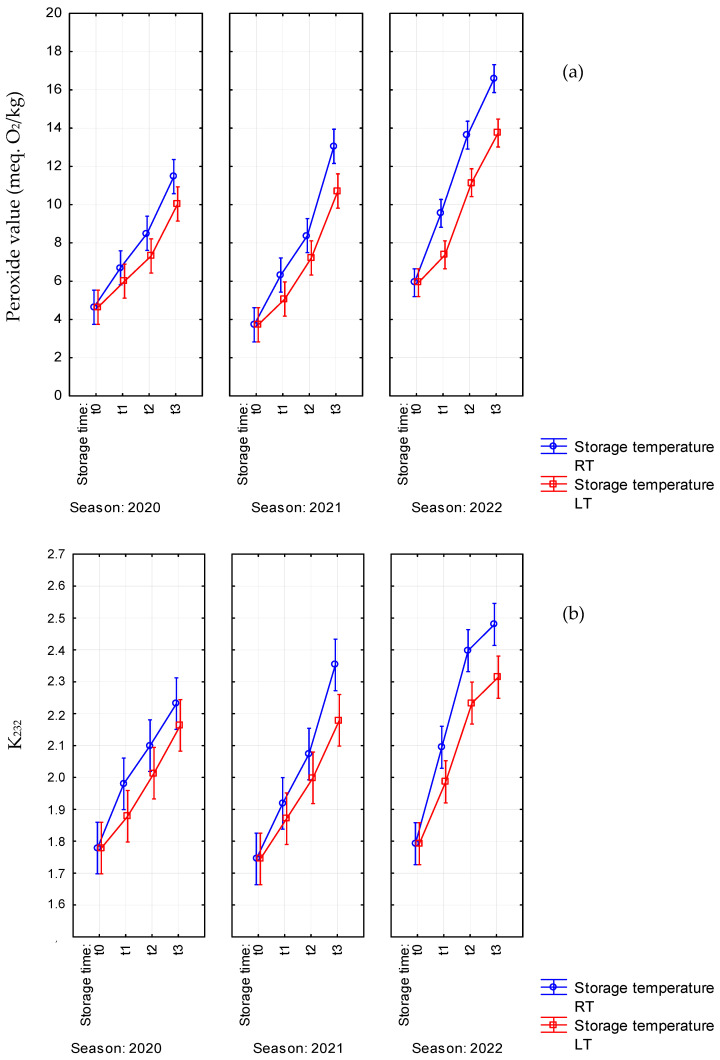
Significant interactions between storage time (t_0_, t_1_: 6 months, t_2_: 12 months, t_3_: 18 months) and storage temperature (RT: room temperature, LT: 4 °C) on (**a**) peroxide value (PV) and (**b**) K_232_ for the EVOO samples of Coratina *cv.* from three consecutive seasons (2020–2022). Vertical bars denote 0.95 confidence intervals.

PV relates to the initial oxidation and allows its detection before the organoleptic perception [23]. In our research, the fresh EVOOs showed low peroxide values (generally under 6 meq. O_2_/kg), indicating good manufacturing conditions according to other literature findings [16,17]; temperature influenced PV, especially in 2022 samples, reaching higher concentrations in oil stored at RT already after 6 months, even though they never exceeded the conventional limit of 20 meq. O_2_/kg. Instead, Mulinacci et al. reported that PV rose to 21.2 meq. O_2_/kg in oil samples after 9 months of storage at RT [14]. However, slower increments in PV were registered when oil samples were stored at LT (Table 1, Figure 1a); these results confirm previous observations about the effect of low storage temperature on the lipid oxidation rate in olive oil [24]. It is worth noting that, contrary to previous reports [11], peroxides formed through the classic autoxidation mechanism did not decrease during the storage of olive oils in the dark without oxygen.

Concerning the spectrophotometric parameters measuring the conjugated forms of unsaturated fatty acids indicative of primary oxidation (K_232_) [25] and secondary oxidation products (K_270_) [26], a significant interaction between storage time and temperature was only registered for K_232_ (F = 3.48, *p* = 0.018), especially in 2021 and 2022, with lower values (around 7%) in samples stored at 4 °C (LT) than room temperature after 18 months (Table 1, Figure 1b). According to previous statements [27], in our conditions, the formation of secondary oxidation products was significantly conditioned by ambient storage temperature (F = 12.45, *p* = 0.0004), while storage at low temperature limited the increase in K_270_ values (Table 1, Figure S1—Supplementary Information). Therefore, storage at 4 °C was overall very effective in preserving the oils from oxidation.

### 3.2. Behavior of Phenolic Compounds of Coratina cv. EVOO During Storage

To assess if and how storage conditions can change the concentration of phenolic compounds and the evolution of their derivatives, EVOO extracts for all storage treatments in the three years were accurately analyzed (Table 2; Figure S2—Supplementary Information).

Even though classifying cultivars as “poor” or “rich” sources of phenols might be misleading, high concentrations of phenolic compounds in Coratina EVOO have been frequently reported in the literature [28,29]. In our study, TPP content of oils at the beginning of the storage period (t_0_) ranged from 860 mg/kg in 2020 to 530 mg/kg in 2022, showing a significant influence of the season factor (F = 92; *p* < 0.001) probably more imputable to the variation in agronomic practices and pedoclimatic conditions than processing technologies [30].

**Figure 2:**
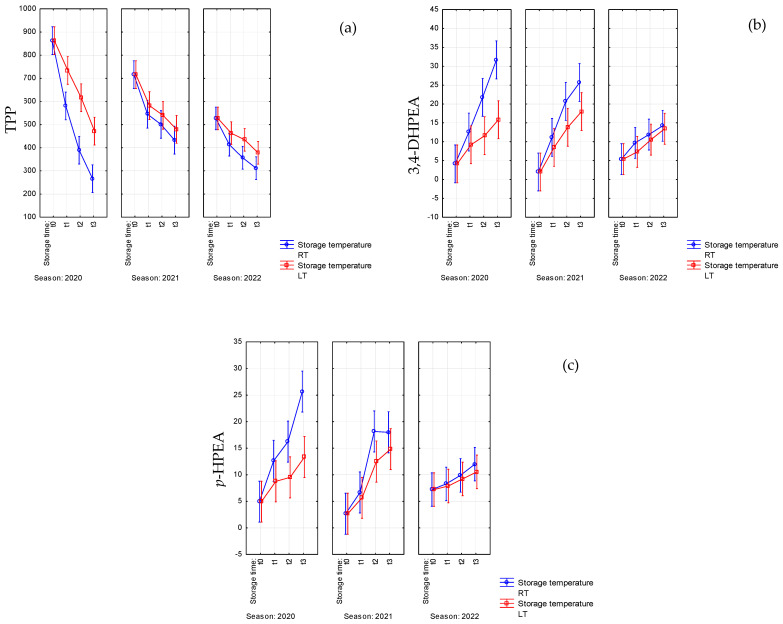
Significant interactions between storage time (t_0_, t_1_: 6 months, t_2_: 12 months, t3: 18 months) and storage temperature (RT: room temperature, LT: 4 °C) on (**a**) total polyphenols (TPPs), (**b**) 3-hydroxytyrosol (3,4-DHPEA), and (**c**) tyrosol (*p*-HPEA) for the EVOO samples of Coratina *cv.* from three consecutive seasons (2020–2022). Vertical bars denote 0.95 confidence intervals.

Polyphenols are recognized for their contribution to the oxidative stability of EVOO, primarily by scavenging free radicals generated from the autoxidation of unsaturated fatty acids [11]. However, these protective compounds are subject to degradation during storage, a process that is markedly accelerated at room temperature relative to freezing conditions [24,28]. Our data confirmed these literature statements; indeed, a positive interaction between storage time and storage temperature (F = 5; *p* = 0.002) was observed. Our Coratina EVOOs already presented a significant reduction in TPP after 6 months (t_1_) of storage, particularly at RT (Table 2, Figure 2). Moreover, according to other research [13,16], it is worth pointing out that the degradation rate of TPP was strongly related to their initial concentration; Coratina EVOOs of 2020, having the highest phenolic content, lost roughly 70% of TPP when stored at RT whilst the oils of 2021 and 2022 lost only 40% of TPP at RT. However, phenols were better preserved at LT (Table 2, Figure 2a).

The concentrations of 3,4-DHPEA and *p*-HPEA increased during storage time, and this trend was significantly influenced by temperature, as evident in the samples of 2020, which showed, after 18 months, doubled values of the simple phenols at RT with respect to LT (Table 2, Figure 2).

The observed increase in hydroxytyrosol and tyrosol is attributed to the partial hydrolysis of secoiridoid compounds [31], even though a significant inverse correlation with the decline in their precursors, 3,4-DHPEA-EA (r = −0.63; N = 48) and p-HPEA-EA (r = −0.32; N = 48), was only evident during the 2020 season (*p* < 0.05). Conversely, in the other two seasons, the concentrations of oleuropein and ligstroside aglycones exhibited a partial increase during storage, independent of temperature. This trend contradicts the expected decrease, which is typically anticipated due to their antioxidant consumption and hydrolytic degradation [15,24,32] (Figure S3—Supplementary Information). The preferential hydrolysis of oleuropein and ligstroside, combined with elevated concentrations of lipophilic antioxidants, protecting them from oxidative reactions, could account for this phenomenon [22]. Nevertheless, more in-depth analyses will be conducted in future studies, including the starting level and degradation rate of lipophilic antioxidants in Coratina *cv.* EVOO, to elucidate the underlying mechanisms.

Concerning oleacin (3,4-DHPEA-EDA) and oleocanthal (*p*-HPEA-EDA), the decarboxymethylated compounds derived from oleuropein and ligstroside, respectively, their concentrations underwent a sharp reduction (tendentially, more consistent at higher storage temperature) reaching down to −78% in 2020 samples at RT after 18 months (Figure S3a,b—Supplementary Information). It is worth remembering that the two secoidiroid derivatives are characterized by the presence of two aldehydic functional groups in an open configuration and possess larger reactivity than monoaldehyde (principally in a closed configuration) oleuropein and ligstroside aglycone [33]; this can explain their marked behavior during storage in all 3 years of study compared to that of the aforementioned secoiridoids (Figure S3a,b vs. Figure S3d,e—Supplementary Information). However, the degradation of 3,4-DHPEA-EDA in the 2020 EVOOs was significantly influenced by the storage temperature (Figure S3d—Supplementary Information). Finally, in agreement with previous literature findings on EVOOs with high polyphenols [16], lignans (pinoresinol + acetoxypinoresinol) strongly dropped during the storage (Figure S3c—Supplementary Information).

### 3.3. Kinetics of Quality and Phenolic Indicators of Coratina cv. EVOO During Storage

Reaction rate constants (k) of qualitative parameters and phenolic compounds of Coratina EVOOs stored at room temperature and 4 °C in the three years of study are listed in Table 3. To avoid an ambiguous interpretation of the kinetic behavior of the indicators in the different samples, it is worth noting that the order of the reactions was assessed through linear regression analysis by plotting the averaged values for each index from all 21 EVOOs against time and visually inspecting the determination coefficients closest to 1 (Figure S4—Supplementary Information).

In agreement with similar research [6,8,32], PV, K_232_, and K_270_ parameters followed an apparent zero-order kinetics, showing high and direct correlations with the storage duration, as confirmed by R^2^ values (>0.98) and the positive slopes of the regression equations. A marked effect of temperature was observed on the rates of lipid oxidation (k_0(PV)_ and k_0(K232)_) and hydroperoxide decomposition (k_0(K270)_); in all samples, the rate increased at room temperature (RT) compared to low-temperature (LT) conditions (Table 3). Regarding polyphenols, as expected [32], the evolution of hydroxytyrosol and tyrosol concentrations during storage time followed a pseudo-zero-order kinetics, with their formation rates from secoiridoid hydrolysis increasing as a function of storage temperature (Figure S4—Supplementary Information, Table 3). The degradation of total phenols, secoiridoids, and lignans followed a second-order kinetics; this finding contrasts with the previous literature on EVOOs stored in both open and closed glass containers, which reported different kinetic models (specifically, pseudo-first-order kinetics). However, our experimental data at both storage temperatures highlighted that the rate of decrease in hydroxytyrosol derivatives (i.e., oleacin) was higher than that of tyrosol derivatives (i.e., oleochantal), confirming how the former are more susceptible to oxidation and thermal decomposition than the latter (Table 3) [32,34,35].

### 3.4. LDA Discriminant Approach to Distinguish EVOO Samples Stored at Different Temperatures

**Figure 3:**
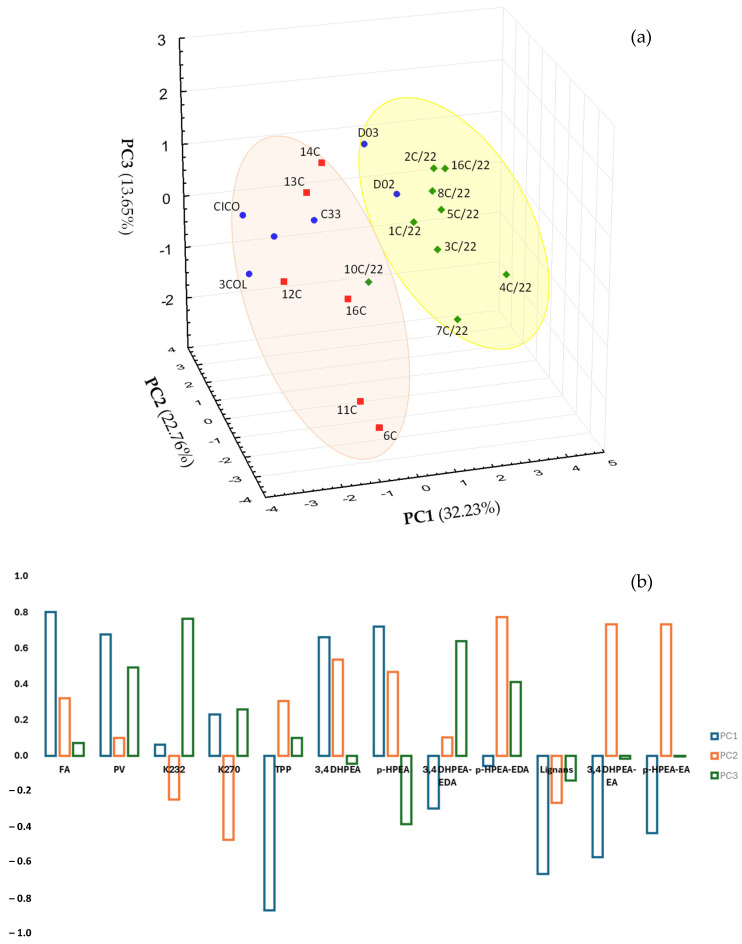
Principal component diagram of 21 EVOO samples (Coratina *cv*.) from three consecutive years: 2020 (blue circles), 2021 (red squares), and 2022 (green diamonds). (**a**) Factor score plots PC1-PC3 account for 68.64% of the total variance explained and (**b**) factor loadings plot. Variables correspond to conventional quality indices and phenolic compounds quantified in oils at t_0_.

At this point, starting from the reaction kinetics listed in Table 3, we wanted to determine which indicators best discriminated among the 21 Coratina *cv*. EVOO samples that were stored at two different temperatures (RT and LT), and which indicators were the best predictors. In order to find out suitable datasets for computing a reliable discriminant function and assessing the predictive utility/validity of the classification function (cross-validation), a PCA was tentatively performed on the qualitative parameters and phenolic compounds analyzed at t_0_ in all EVOOs of the three years of study (Figure 3). The factor score plot accounting for 68.64% of total explained variance (Figure 3a) evidenced the clustering of the Coratina samples belonging to 2020 and 2021 differentiated from 2022 samples mainly due to FA and TPP, K_232_, and *p*-HPEA-EDA, having the highest factor loadings (>|0.8|) on PC1, PC2, and PC3, respectively (Figure 3b). Definitely, k-values of 2020 and 2021 were used for obtaining discriminant and classification functions, while 2022 k-values were employed to cross-validate the predictive classification of samples according to the storage temperature.

The discriminant function was obtained by selecting a forward stepwise analysis, which capitalizes on chance because it picks and chooses the k-variables to be included in the model to yield the maximum discrimination. F-to-enter and F-to-remove values were set to 1 and 0, respectively, for entering all variables to evaluate in the stepwise analysis; moreover, tolerance was maintained at 0.01 to exclude the completely redundant variables from the model, but avoiding round-off errors, which can lead to unstable estimates of parameters of the model. The final discriminant function included four k-variables, specifically k_2(p-HPEA-EDA)_, k_0(3,4-DHPEA)_, k_0(PV)_, and k_2(p-HPEA-EA)_, contributing most to the overall discrimination as demonstrated by partial Wilks’ Lambda (the smaller this parameter, the greater is the contribution to the overall discrimination) and F values (Table S2—Supplementary Information). Thus, considering the raw coefficients, the obtained discriminant function can be expressed as follows:

(4) $$Y=6.4-58584.7k_{2\left(p-HPEA-EDA\right)}-48.1k_{0\left(3,4-DHPEA\right)}-229.5k_{0\left(PV\right)}+469.7\;k_{2(p-HPEA-EA)}$$

To summarize the findings so far, it appeared that significant discrimination (*p* = 0.000012) existed among Coratina *cv.* EVOOs stored at different temperatures, which was inversely proportional to the rate of formation of oleocanthal (k_2(p-HPEA-EDA)_), hydroxytyrosol (k_0(3,4-DHPEA)_), and hydroperoxides (k_0(PV)_), and directly proportional to the degradation of ligstroside (k_2(p-HPEA-EA)_).

Subsequently, to verify if the discriminant k-variables efficiently worked in classifying the oil samples into the right RT or LT group, two classification functions were computed for each group:

(5) $$S_{RT}=-35.2+429873.2{*k}_{2\left(p-HPEA-EDA\right)}+370.7{*k}_{0\left(3,4-DHPEA\right)}+2269.6*k_{0\left(PV\right)}-3674.8{*k}_{2(p-HPEA-EA)}$$

(6) $$S_{LT}=-13.7+233172.8*k_{2\left(p-HPEA-EDA\right)}+209.2*k_{0\left(3,4-DHPEA\right)}+1498.5*k_{0\left(PV\right)}-2096.8{*k}_{2(p-HPEA-EA)}$$

As discussed above, oil samples from the 2022 season were used to try out the predictive power of the functions; because the samples were classified into the group for which they had the highest classification score (S_RT_ or S_LT_), a total percentage of 88.89% of correct attribution was observed, with 7/9 EVOOs correctly assigned to the RT group and 9/9 EVOOs correctly assigned to the LT group (Table 4).

## 4. Conclusions

Extra-virgin olive oil is a cornerstone of healthy dietary patterns, notably in the Mediterranean diet, owing to its rich composition of monounsaturated fatty acids along with bioactive minor compounds like polyphenols, which exhibit well-documented health benefits. The qualitative and quantitative profile of EVOO can vary significantly depending on factors such as cultivar, harvest timing, processing techniques, and storage conditions. In the present study, the kinetic evolution of qualitative parameters in 21 Coratina *cv.* EVOOs was monitored during 18 months of real-time storage under dark conditions, in order to identify key variables—including fatty acids (FAs), peroxide value (PV), UV absorbance (K_232_, K_270_, ΔK), simple phenols, lignans, and secoiridoids—that reliably discriminate EVOOs based on storage temperature (RT vs. LT), using a multivariate discriminant approach (MANOVA, PCA, and LDA).

From gathered results regarding the different kinetic evolution of qualitative parameters and polyphenols, the storage at 4 °C appeared as the most effective in preserving the quality of the studied EVOOs, by reducing the oxidation to hydroperoxides and the degradation of phenolic compounds with respect to RT. Moreover, employing k-values from 2020 and 2021 to establish classification functions and 2022 k-values for cross-validation to predict sample classification, a highly significant discrimination (*p* = 0.000012) was observed among EVOOs stored at different temperatures. This discrimination was inversely proportional to the formation rate of oleocanthal (*k*_2*(p-HPEA-EDA)*_), hydroxytyrosol (k_0(3,4-DHPEA)_), and hydroperoxides (*k*_0*(PV)*_), while directly proportional to the degradation of ligstroside (*k*_2*(p-HPEA-EA)*_). Therefore, it can be concluded these discriminant k-variables effectively classified oil samples into the correct RT or LT groups, achieving an overall correct attribution rate of ~90%.

## Figures and Tables

**Table 1:** Conventional quality indices, reported as mean (m) ± standard deviation (σ), for 21 olive oils Coratina cv. from three consecutive seasons.

Seasons			2020	2021	2022
Samples/ Parameters			3COL, CICO, P18, C33, D02, D03	6-C, 11-C, 12-C, 13-C, 14-C, 16-C	1C/22, 2C/22, 3C/22, 4C/22, 5C/22, 7C/22, 8C/22, 10C/22, 16C/22
**FA**			**m ± σ**	**m ± σ**	**m ± σ**
	RT	t_0_	0.22 ± 0.02	0.18 ± 0.03	0.26 ± 0.05
		t_1_	0.218 ± 0.018	0.18 ± 0.03	0.27 ± 0.05
		t_2_	0.222 ± 0.019	0.18 ± 0.03	0.27 ± 0.05
		t_3_	0.23 ± 0.02	0.17 ± 0.03	0.28 ± 0.06
	LT	t_0_	0.22 ± 0.02	0.18 ± 0.03	0.26 ± 0.05
		t_1_	0.22 ± 0.02	0.18 ± 0.03	0.27 ± 0.05
		t_2_	0.22 ± 0.02	0.18 ± 0.03	0.27 ± 0.05
		t_3_	0.22 ± 0.02	0.18 ± 0.03	0.27 ± 0.06
**PV**	RT	t_0_	4.6 ± 0.8 e	3.7 ± 0.7 e	5.9 ± 1.0 e
		t_1_	6.7 ± 1.1 cde	6.3 ± 0.5 cd	9.5 ± 0.8 c
		t_2_	8.5 ± 1.3 bc	8.4 ± 0.6 c	13.6 ± 1.6 b
		t_3_	11.5 ± 1.7 a	13.1 ± 0.5 a	16.6 ± 1.1 a
	LT	t_0_	4.6 ± 0.8 e	3.7 ± 0.7 e	5.9 ± 1.0 e
		t_1_	6.0 ± 1.3 de	5.1 ± 0.3 de	7.4 ± 1.0 de
		t_2_	7.3 ± 1.4 cd	7.2 ± 0.7 cd	11.1 ± 1.0 c
		t_3_	10 ± 2 ab	10.7 ± 1.4 b	13.7 ± 0.8 b
**K_232_**	RT	t_0_	1.78 ± 0.12 d	1.74 ± 0.11 d	1.79 ± 0.09 e
		t_1_	1.98 ± 0.17 bcd	1.92 ± 0.06 cd	2.09 ± 0.06 cd
		t_2_	2.10 ± 0.17 ab	2.07 ± 0.05 bc	2.40 ± 0.08 ab
		t_3_	2.23 ± 0.18 a	2.35 ± 0.12 a	2.48 ± 0.07 a
	LT	t_0_	1.78 ± 0.12 d	1.74 ± 0.11d	1.79 ± 0.09 e
		t_1_	1.88 ± 0.10 cd	1.87 ± 0.09 cd	1.99 ± 0.05 d
		t_2_	2.01 ± 0.12 bc	2.00 ± 0.05 bc	2.23 ± 0.05bc
		t_3_	2.16 ± 0.16 ab	2.18 ± 0.05 ab	2.31 ± 0.03 ab
**K_270_**	RT	t_0_	0.134 ± 0.017	0.153 ± 0.008	0.143 ± 0.010
		t_1_	0.16 ± 0.03	0.163 ± 0.009	0.175 ± 0.008
		t_2_	0.17 ± 0.03	0.180 ± 0.011	0.211 ± 0.011
		t_3_	0.19 ± 0.03	0.21 ± 0.03	0.222 ± 0.008
	LT	t_0_	0.134 ± 0.017	0.153 ± 0.008	0.143 ± 0.010
		t_1_	0.15 ± 0.02	0.158 ± 0.007	0.159 ± 0.007
		t_2_	0.17 ± 0.02	0.169 ± 0.008	0.192 ± 0.016
		t_3_	0.19 ± 0.02	0.19 ± 0.02	0.214 ± 0.010
**ΔK**	RT	t_0_	−0.001 ± 0.003	−0.002 ± 0.001	−0.002 ± 0.002
		t_1_	0.001 ± 0.001	0.000 ± 0.001	0.000 ± 0.001
		t_2_	0.001 ± 0.001	0.001 ± 0.001	0.001 ± 0.001
		t_3_	0.002 ± 0.000	0.002 ± 0.001	0.001 ± 0.001
	LT	t_0_	−0.001 ± 0.003	−0.002 ± 0.001	−0.002 ± 0.002
		t_1_	0.001 ± 0.002	−0.001 ± 0.001	−0.001 ± 0.002
		t_2_	0.001 ± 0.001	−0.002 ± 0.004	0.000 ± 0.001
		t_3_	0.002 ± 0.001	0.002 ± 0.001	0.001 ± 0.001

FA = free acidity (% oleic acid); PV = peroxide value (meq. O_2_/kg). The mean and standard deviation were calculated from parameter measurements of all samples for each season. Different letters in the same column for each parameter and season indicate significant difference at 5% level (Tukey HSD test).

**Table 2:** Content of phenolic compounds (mg/kg), reported as mean (m) ± standard deviation (σ), in 21 Coratina *cv.* extra-virgin olive oils from three consecutive seasons.

Season			2020	2021	2022
Samples/ Compounds			3COL, CICO, P18, C33, D02, D03	6-C, 11-C, 12-C, 13-C, 14-C, 16-C	1C/22, 2C/22, 3C/22, 4C/22, 5C/22, 7C/22, 8C/22, 10C/22, 16C/22
**TPP**	RT	t_0_	860 ± 140 a	720 ± 80 a	530 ± 70 a
		t_1_	580 ± 30 bc	550 ± 60 b	410 ± 70 abc
		t_2_	390 ± 70 d	500 ± 70 b	360 ± 60 bc
		t_3_	270 ± 30 d	430 ± 80 b	300 ± 40 c
	LT	t_0_	860 ± 140 a	720 ± 80 a	530 ± 70 a
		t_1_	730 ± 50 ab	580 ± 70 b	460 ± 80 ab
		t_2_	620 ± 80 bc	540 ± 70 b	430 ± 80 abc
		t_3_	470 ± 50 c	480 ± 80 b	380 ± 60 bc
**3,4-DHPEA**	RT	t_0_	4 ± 2 c	2.0 ± 0.9 c	5 ± 2
		t_1_	13 ± 3 bc	11 ± 10 bc	10 ± 5
		t_2_	22 ± 4 ab	21 ± 16 ab	12 ± 6
		t_3_	32 ± 5 a	26 ± 14 a	14 ± 5
	LT	t_0_	4 ± 2 c	2.0 ± 0.9 c	5 ± 2
		t_1_	9 ± 3 bc	9 ± 7 bc	7 ± 4
		t_2_	12 ± 4 bc	14 ± 9 abc	11 ± 5
		t_3_	16 ± 3 bc	18 ± 9 ab	13 ± 5
***p*-HPEA**	RT	t_0_	5 ± 2 c	2.7 ± 1.8 c	7 ± 3
		t_1_	12.6 ± 1.8 bc	7 ± 5 bc	8 ± 3
		t_2_	16 ± 3 ab	18 ± 14 a	10 ± 6
		t_3_	26 ± 4 a	18 ± 10 a	12 ± 4
	LT	t_0_	5 ± 2 c	2.7 ± 1.8 c	7 ± 3
		t_1_	9 ± 4 bc	6 ± 3 bc	8 ± 2
		t_2_	10 ± 4 bc	13 ± 9 abc	9 ± 2
		t_3_	13 ± 3 bc	15 ± 6 ab	10 ± 3
**3,4-DHPEA-EDA**	RT	t_0_	180 ± 70	150 ± 70	130 ± 40
		t_1_	100 ± 40	90 ± 60	90 ± 20
		t_2_	60 ± 30	70 ± 40	80 ± 30
		t_3_	39 ± 19	60 ± 40	60 ± 20
	LT	t_0_	180 ± 70	150 ± 70	130 ± 40
		t_1_	170 ± 60	100 ± 60	110 ± 30
		t_2_	110 ± 50	90 ± 50	110 ± 30
		t_3_	90 ± 40	70 ± 40	80 ± 20
***p*-HPEA-EDA**	RT	t_0_	180 ± 40	120 ± 60	150 ± 20
		t_1_	110 ± 40	80 ± 30	100 ± 17
		t_2_	58 ± 14	60 ± 30	90 ± 20
		t_3_	40 ± 11	50 ± 30	67 ± 15
	LT	t_0_	180 ± 40	120 ± 60	150 ± 20
		t_1_	140 ± 30	90 ± 40	130 ± 20
		t_2_	100 ± 40	70 ± 40	118 ± 18
		t_3_	90 ± 30	70 ± 40	89 ± 13
**Lignans**	RT	t_0_	110 ± 30	100 ± 40	70 ± 30
		t_1_	83 ± 19	60 ± 18	40 ± 20
		t_2_	52 ± 10	42 ± 13	33 ± 14
		t_3_	39 ± 10	36 ± 10	28 ± 10
	LT	t_0_	110 ± 30	100 ± 40	70 ± 30
		t_1_	100 ± 30	70 ± 20	50 ± 30
		t_2_	70 ± 40	60 ± 20	50 ± 20
		t_3_	60 ± 40	50 ± 20	38 ± 18
**3,4-DHPEA-EA**	RT	t_0_	110 ± 50	83 ± 19	74 ± 12
		t_1_	68 ± 16	140 ± 60	70 ± 30
		t_2_	48 ± 14	170 ± 70	70 ± 30
		t_3_	30 ± 17	160 ± 60	70 ± 20
	LT	t_0_	110 ± 50	83 ± 19	74 ± 12
		t_1_	110 ± 40	140 ± 70	70 ± 30
		t_2_	110 ± 30	170 ± 70	80 ± 30
		t_3_	90 ± 20	150 ± 50	80 ± 30
***p*-HPEA-EA**	RT	t_0_	38 ± 15	33 ± 14	27 ± 5
		t_1_	35 ± 14	40 ± 30	36 ± 10
		t_2_	27 ± 20	70 ± 50	40 ± 12
		t_3_	14 ± 3	70 ± 40	45 ± 11
	LT	t_0_	38 ± 15	33 ± 14	27 ± 5
		t_1_	35 ± 13	40 ± 30	36 ± 13
		t_2_	42 ± 20	80 ± 30	43 ± 16
		t_3_	26 ± 12	80 ± 30	52 ± 14

The mean and standard deviation were calculated from compound measurements of all samples for each season. Different letters in the same column for each compound and season indicate significant difference at 5% level (Tukey HSD test). TPP = total polyphenols, 3,4-DHPEA = 3-hydroxytyrosol, p-HPEA = tyrosol, 3,4-DHPEA-EDA = oleacein, *p*-HPEA-EDA = oleocanthal, lignans = pinoresinol + acetoxypinoresinol, 3,4-DHPEA-EA = oleuropein aglycone, and p-HPEA-EA = ligstroside aglycone.

**Table 3:** Reaction rate constants (k) of qualitative and phenolic parameters of Coratina EVOOs stored at room temperature (RT) and 4 °C (LT).

EVOOs	Year	T	k_0(PV)_ (meqO_2_kg^−1^day^−1^)	k_0(K232)_ (D.O. day^−1^)	k_0(K270)_ (D.O. day^−1^)	k_2(TPP)_ (kg mg^−1^ day^−1^)	k_0(3,4 DHPEA)_ (mg kg^−1^ day^−1^)	k_0(p-HPEA)_ (mg kg^−1^ day^−1^)	k_2(3,4 DHPEA-EDA)_ (kg mg^−1^ day^−1^)	k_2(p-HPEA-EDA)_ (kg mg^−1^ day^−1^)	k_2(lignans)_ (kg mg^−1^ day^−1^)	k_2(3,4 DHPEA-EA)_ (kg mg^−1^ day^−1^)	k_2(p-HPEA-EA)_ (kg mg^−1^ day^−1^)
3COL	2020	RT	0.0102	7.1 × 10^−4^	1.0 × 10^−4^	4.8 × 10^−6^	0.0577	0.0438	4.0 × 10^−5^	5.0 × 10^−5^	1.8 × 10^−5^	−3.1 × 10^−3^	−2.1 × 10^−3^
CICO	2020	RT	0.0098	5.7 × 10^−4^	9.2 × 10^−5^	4.5 × 10^−6^	0.0344	0.0213	6.4 × 10^−5^	3.7 × 10^−5^	2.5 × 10^−5^	−1.7 × 10^−3^	−2.4 × 10^−3^
P18	2020	RT	0.0132	7.2 × 10^−4^	1.1 × 10^−4^	4.4 × 10^−6^	0.0512	0.0402	2.0 × 10^−5^	2.5 × 10^−5^	4.8 × 10^−5^	−2.4 × 10^−3^	−1.9 × 10^−3^
C33	2020	RT	0.0149	11 × 10^−4^	8.2 × 10^−5^	3.9 × 10^−6^	0.0537	0.0379	2.3 × 10^−5^	3.0 × 10^−5^	3.4 × 10^−5^	−3.2 × 10^−3^	−2.3 × 10^−3^
D02	2020	RT	0.0169	12 × 10^−4^	1.5 × 10^−4^	5.8 × 10^−6^	0.052	0.0356	5.8 × 10^−5^	4.7 × 10^−5^	2.9 × 10^−5^	−1.6 × 10^−3^	−3.0 × 10^−4^
D03	2020	RT	0.0092	6.5 × 10^−4^	8.5 × 10^−5^	5.6 × 10^−6^	0.0567	0.0418	6.8 × 10^−5^	4.1 × 10^−5^	3.7 × 10^−5^	−2.8 × 10^−3^	−1.4 × 10^−3^
3COL	2020	LT	0.0071	4.1 × 10^−4^	9.1 × 10^−5^	1.9 × 10^−6^	0.0289	0.0145	1.2 × 10^−5^	1.8 × 10^−5^	5.7 × 10^−6^	−4.0 × 10^−4^	−5.0 × 10^−4^
CICO	2020	LT	0.0062	4.7 × 10^−4^	8.2 × 10^−5^	1.5 × 10^−6^	0.0212	0.0108	4.2 × 10^−5^	4.0 × 10^−5^	-7.0 × 10^−7^	−6.0 × 10^−4^	−1.0 × 10^−4^
P18	2020	LT	0.009	7.8 × 10^−4^	1.2 × 10^−4^	2.5 × 10^−6^	0.016	0.0114	1.2 × 10^−5^	1.3 × 10^−5^	3.0 × 10^−5^	−5.0 × 10^−5^	−6.0 × 10^−4^
C33	2020	LT	0.015	9.6 × 10^−4^	9.4 × 10^−5^	1.2 × 10^−6^	0.0128	0.0054	7.0 × 10^−6^	7.0 × 10^−6^	7.2 × 10^−5^	−7.0 × 10^−4^	−2.0 × 10^−3^
D02	2020	LT	0.012	10 × 10^−4^	1.2 × 10^−4^	1.6 × 10^−6^	0.0248	0.0216	6.0 × 10^−6^	4.0 × 10^−6^	2.7 × 10^−5^	7.0 × 10^−4^	−4.0 × 10^−4^
D03	2020	LT	0.0089	7.0 × 10^−4^	9.1 × 10^−5^	1.8 × 10^−6^	0.0223	0.0237	9.0 × 10^−6^	3.0 × 10^−6^	3.4 × 10^−5^	−4.0 × 10^−4^	−7.0 × 10^−4^
6C	2021	RT	0.0138	13 × 10^−4^	8.0 × 10^−5^	8.6 × 10^−7^	0.034	0.021	6.3 × 10^−5^	6.5 × 10^−5^	3.3 × 10^−5^	2.4 × 10^−3^	3.6 × 10^−3^
11C	2021	RT	0.0216	13 × 10^−4^	2.0 × 10^−4^	2.4 × 10^−6^	0.0205	0.0161	4.4 × 10^−5^	7.7 × 10^−5^	7.9 × 10^−5^	1.6 × 10^−3^	2.2 × 10^−3^
12C	2021	RT	0.0174	12 × 10^−4^	8.0 × 10^−5^	2.0 × 10^−6^	0.0885	0.0633	2.7 × 10^−5^	1.3 × 10^−5^	4.7 × 10^−5^	5.0 × 10^−4^	−3.0 × 10^−5^
13C	2021	RT	0.0183	12 × 10^−4^	1.0 × 10^−4^	2.3 × 10^−6^	0.0598	0.0204	1.7 × 10^−5^	2.4 × 10^−5^	2.4 × 10^−5^	4.0 × 10^−4^	1.6 × 10^−3^
14C	2021	RT	0.0168	8.0 × 10^−4^	9.0 × 10^−5^	1.1 × 10^−6^	0.0644	0.0642	7.7 × 10^−6^	2.4 × 10^−5^	2.0 × 10^−5^	3.0 × 10^−4^	7.0 × 10^−4^
16C	2021	RT	0.0189	12 × 10^−4^	1.0 × 10^−4^	2.1 × 10^−6^	0.0153	0.0172	1.7 × 10^−5^	2.5 × 10^−5^	2.6 × 10^−5^	1.7 × 10^−3^	1.4 × 10^−3^
6C	2021	LT	0.0131	10 × 10^−4^	7.0 × 10^−5^	7.1 × 10^−7^	0.024	0.0247	6.3 × 10^−5^	1.0 × 10^−5^	1.6 × 10^−5^	2.2 × 10^−3^	3.2 × 10^−3^
11C	2021	LT	0.0173	10 × 10^−4^	1.0 × 10^−4^	1.6 × 10^−6^	0.0156	0.0143	3.8 × 10^−5^	2.4 × 10^−5^	5.7 × 10^−5^	1.5 × 10^−3^	3.1 × 10^−3^
12C	2021	LT	0.0116	11 × 10^−4^	9.0 × 10^−5^	2.0 × 10^−6^	0.0552	0.0399	1.4 × 10^−5^	1.0 × 10^−5^	3.5 × 10^−5^	5.0 × 10^−5^	−9.0 × 10^−5^
13C	2021	LT	0.0167	8.0 × 10^−4^	8.0 × 10^−5^	1.9 × 10^−6^	0.0345	0.0176	1.0 × 10^−5^	1.0 × 10^−5^	1.6 × 10^−5^	2.0 × 10^−4^	7.0 × 10^−4^
14C	2021	LT	0.0083	5.0 × 10^−4^	8.0 × 10^−5^	8.3 × 10^−7^	0.046	0.0435	5.7 × 10^−6^	1.0 × 10^−5^	1.4 × 10^−5^	7.0 × 10^−4^	1.9 × 10^−3^
16C	2021	LT	0.0156	6.0 × 10^−4^	8.0 × 10^−5^	1.1 × 10^−6^	0.0118	0.013	8.9 × 10^−6^	1.4 × 10^−5^	8.0 × 10^−6^	1.8 × 10^−3^	2.3 × 10^−3^
1C/22	2022	RT	0.0242	13 × 10^−4^	1.4 × 10^−4^	2.1 × 10^−6^	0.0098	0.0056	4.9 × 10^−6^	6.0 × 10^−6^	1.7 × 10^−5^	−3.2 × 10^−4^	−1.1 × 10^−4^
2C/22	2022	RT	0.0253	17 × 10^−4^	2.3 × 10^−4^	3.4 × 10^−6^	0.0111	0.0158	1.0 × 10^−5^	8.9 × 10^−6^	4.0 × 10^−5^	−1.4 × 10^−4^	8.0 × 10^−5^
3C/22	2022	RT	0.0229	16 × 10^−4^	1.4 × 10^−4^	3.6 × 10^−6^	0.0203	0.0071	3.9 × 10^−5^	2.2 × 10^−5^	3.1 × 10^−5^	−3.7 × 10^−4^	1.6 × 10^−3^
4C/22	2022	RT	0.0216	16 × 10^−4^	1.4 × 10^−4^	2.3 × 10^−6^	0.0066	−0.0086	1.5 × 10^−5^	1.0 × 10^−5^	4.9 × 10^−6^	−3.3 × 10^−4^	7.7 × 10^−4^
5C/22	2022	RT	0.0254	16 × 10^−4^	1.9 × 10^−4^	2.7 × 10^−6^	0.0134	0.0056	1.2 × 10^−5^	1.5 × 10^−5^	7.1 × 10^−5^	1.3 × 10^−4^	6.5 × 10^−4^
7C/22	2022	RT	0.0265	17 × 10^−4^	2.0 × 10^−4^	3.4 × 10^−6^	0.022	0.0017	5.6 × 10^−5^	2.2 × 10^−5^	6.4 × 10^−5^	2.4 × 10^−4^	1.2 × 10^−3^
8C/22	2022	RT	0.0243	14 × 10^−4^	1.8 × 10^−4^	3.1 × 10^−6^	0.0184	0.0064	1.4 × 10^−5^	2.2 × 10^−5^	6.2 × 10^−5^	1.0 × 10^−4^	8.9 × 10^−4^
10C/22	2022	RT	0.0256	14 × 10^−4^	1.9 × 10^−4^	3.3 × 10^−6^	0.0241	0.0218	2.3 × 10^−5^	2.6 × 10^−5^	3.0 × 10^−5^	4.3 × 10^−4^	1.8 × 10^−3^
16C/22	2022	RT	0.0152	14 × 10^−4^	1.6 × 10^−4^	1.8 × 10^−6^	0.0405	0.0388	1.9 × 10^−5^	1.7 × 10^−5^	7.9 × 10^−5^	9.0 × 10^−5^	1.1 × 10^−3^
1C/22	2022	LT	0.0183	11 × 10^−4^	1.1 × 10^−4^	9.0 × 10^−7^	0.0268	0.018	5.9 × 10^−6^	9.1 × 10^−6^	2.1 × 10^−5^	6.6 × 10^−5^	−9.8 × 10^−5^
2C/22	2022	LT	0.0163	10 × 10^−4^	1.7 × 10^−4^	2.3 × 10^−6^	0.0091	0.0066	9.6 × 10^−6^	7.5 × 10^−6^	3.9 × 10^−5^	−2.3 × 10^−4^	5.3 × 10^−4^
3C/22	2022	LT	0.0198	13 × 10^−4^	1.5 × 10^−4^	2.4 × 10^−6^	0.013	0.0061	5.7 × 10^−6^	1.2 × 10^−5^	2.1 × 10^−5^	−3.8 × 10^−4^	1.1 × 10^−3^
4C/22	2022	LT	0.0208	15 × 10^−4^	1.6 × 10^−4^	1.6 × 10^−6^	0.014	−0.0029	6.9 × 10^−6^	7.5 × 10^−6^	-5.6 × 10^−6^	5.9 × 10^−4^	1.1 × 10^−3^
5C/22	2022	LT	0.019	11 × 10^−4^	1.5 × 10^−4^	1.2 × 10^−6^	0.0114	0.0106	9.0 × 10^−6^	8.3 × 10^−6^	4.5 × 10^−6^	2.6 × 10^−4^	9.6 × 10^−4^
7C/22	2022	LT	0.0197	13 × 10^−4^	1.6 × 10^−4^	2.0 × 10^−6^	0.0148	−0.0037	2.1 × 10^−5^	1.5 × 10^−5^	4.7 × 10^−5^	3.6 × 10^−4^	1.6 × 10^−3^
8C/22	2022	LT	0.0166	12 × 10^−4^	1.7 × 10^−4^	1.2 × 10^−6^	0.0103	0.0026	1.0 × 10^−5^	7.5 × 10^−6^	2.4 × 10^−5^	3.4 × 10^−4^	1.4 × 10^−3^
10C/22	2022	LT	0.0192	12 × 10^−4^	2.5 × 10^−4^	1.3 × 10^−6^	0.0282	0.013	1.3 × 10^−5^	4.6 × 10^−6^	8.4 × 10^−6^	8.5 × 10^−4^	2.2 × 10^−3^
16C/22	2022	LT	0.011	9.0 × 10^−4^	1.2 × 10^−4^	1.7 × 10^−6^	0.0337	0.0169	2.3 × 10^−5^	1.0 × 10^−5^	5.7 × 10^−5^	5.0 × 10^−4^	2.1 × 10^−3^

k_0_: pseudo zero-order kinetics; k_2_: second-order kinetics.

**Table 4:** Classification scores of Coratina EVOOs from the 2022 season.

	Observed Classification	Predicted Classification	
		RT *p* = 0.50	LT *p* = 0.50
1C/22	RT	26.34 ^a^	26.24 ^a^
2C/22	RT	29.87	28.44
3C/22	RT	27.96	26.68
4C/22	RT	17.83	20.81
5C/22	RT	31.43	29.28
7C/22	RT	38.19	33.24
8C/22	RT	32.74	29.71
10C/22	RT	36.35	31.97
16C/22	RT	17.45	19.14
1C/22	LT	20.54	21.66
2C/22	LT	6.44	13.27
3C/22	LT	15.63	19.16
4C/22	LT	16.38	19.84
5C/22	LT	12.19	17.08
7C/22	LT	15.69	19.13
8C/22	LT	4.37	12.14
10C/22	LT	12.72	17.43
16C/22	LT	−0.99	7.86

^a^ S_RT_ and S_LT_ classification score calculated through the classification functions (Equations (5) and (6), respectively).

## Data Availability

Data is contained within the article and Supplementary Materials; while raw kinetic data will be made available.

## Supplementary Materials

The following supporting information can be downloaded at https://www.mdpi.com/article/10.3390/antiox14111379/s1, Figure S1: Not significant interactions between storage time (t_0_, t_1_: 6 months, t_2_: 12 months, t_3_: 18 months) and storage temperature (RT: room temperature, LT: 4 °C) on free acidity (a) and K_270_ (b) for the EVOO samples of Coratina *cv.* from three consecutive seasons (2020–2022); Figure S2: HPLC-DAD chromatograms registered at 280 nm of Coratina *cv.* from 2020 season; Figure S3: Non-significant interactions between storage time (t_0_, t_1_: 6 months, t_2_: 12 months, t_3_: 18 months) and storage temperature (RT: room temperature, LT: 4 °C) on (a) oleacein (3,4-DHPEA-EDA), (b) oleocanthal (p-HPEA-EDA), (c) lignans, (d) oleuropein aglycone (3,4-DHPEA-EA), and (e) ligstroside aglycone (p-HPEA-EA) for the EVOO samples of Coratina *cv.* from three consecutive seasons (2020–2022); Figure S4: Kinetics of quality and phenolic indicators, averaged along the three years, of Coratina *cv*. EVOO during storage time; Table S1: Univariate results of full factorial two-way MANOVA analysis; Table S2: Discriminant function parameters to review the independent contributions for each k-variable to the overall discrimination between Coratina EVOOs stored at room temperature (RT) and 4 °C (LT).
