# Supplementary material for: Effect of Long-Term Storage Temperature on the Quality of Extra-Virgin Olive Oil (Coratina cv.): A Multivariate Discriminant Approach
Source: Antioxidants (Basel). 2025 Nov 19;14(11):1379. doi: 10.3390/antiox14111379 (PMC12649587; doi:10.3390/antiox14111379)
Supplement: Supplementary file 1 [file antioxidants-14-01379-s001.zip › Figure S1.pdf]

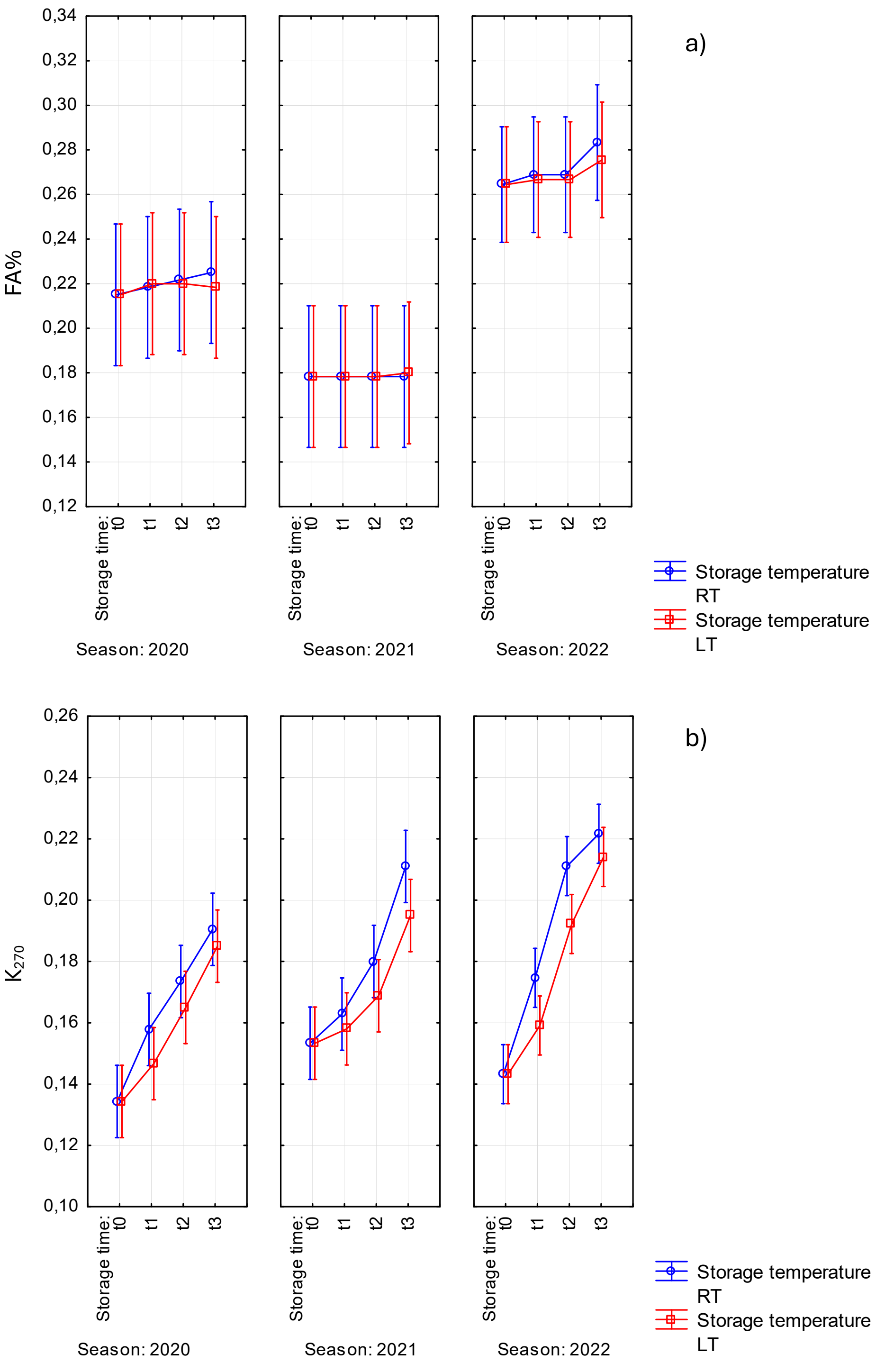

Figure S1. Not significant interactions between storage time (t_0, t_1: 6 months, t_2: 12 months, t_3: 18 months) and storage temperature (RT: room temperature, LT: 4 °C) on free acidity (a) and K_{270} (b) for the EVOO samples of Coratina *cv.* from three consecutive seasons (2020 – 2022). Vertical bars denote 0.95 confidence intervals.
