# Supplementary material for: Effect of Long-Term Storage Temperature on the Quality of Extra-Virgin Olive Oil (Coratina cv.): A Multivariate Discriminant Approach
Source: Antioxidants (Basel). 2025 Nov 19;14(11):1379. doi: 10.3390/antiox14111379 (PMC12649587; doi:10.3390/antiox14111379)
Supplement: Supplementary file 1 [file antioxidants-14-01379-s001.zip › Figure S2-2.pdf]

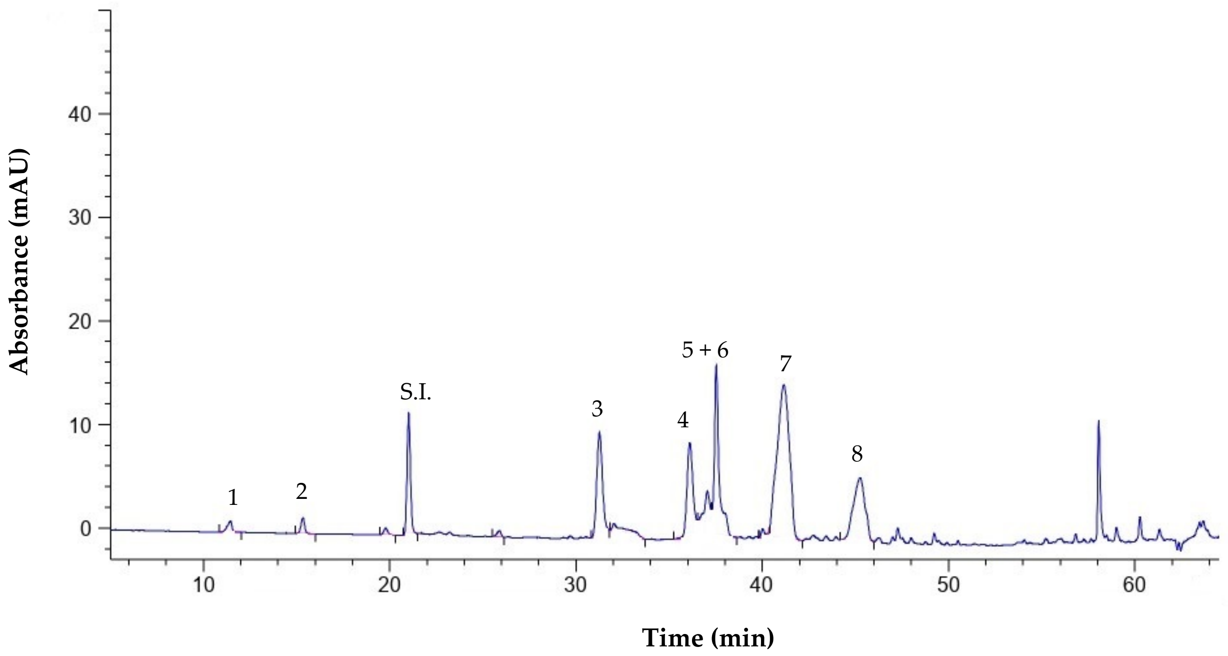

Figure S2. HPLC-DAD chromatograms registered at 280 nm of Coratina *cv.* from 2020 season. 1) 3-hydroxytyrosol (3,4 – DHPEA) (RRT* = 0.58), 2) tyrosol (*p*-HPEA) (RRT = 0.77), 3) oleacin (3,4 – DHPEA-EDA) (RTT = 1.48), 4) oleocanthal (*p*-HPEA-EDA) (RRT = 1.70), 5 + 6) lignans (pinoresinol + acetoxypinoresinol) (RRT = 1.76), 7) oleuropein-aglycone (3,4 – DHPEA-EA) (RRT = 1.90), 8) ligstroside-aglycone (*p*-HPEA-EA) (RRT = 2.06). S.I. = syringic acid.

*Relative retention time calculated with respect to the retention time of internal standard.
