# Supplementary material for: Effect of Long-Term Storage Temperature on the Quality of Extra-Virgin Olive Oil (Coratina cv.): A Multivariate Discriminant Approach
Source: Antioxidants (Basel). 2025 Nov 19;14(11):1379. doi: 10.3390/antiox14111379 (PMC12649587; doi:10.3390/antiox14111379)
Supplement: Supplementary file 1 [file antioxidants-14-01379-s001.zip › Figure S4.pdf]

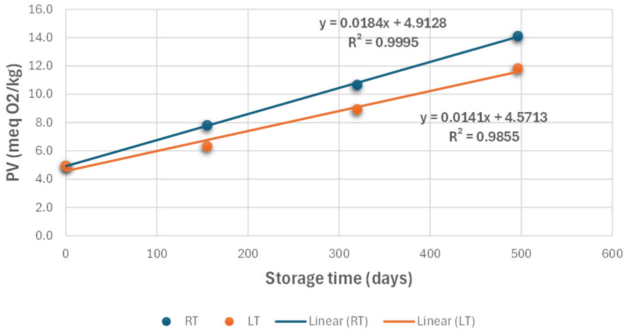
PV (meq O2/kg)
Storage time (days)
y = 0.0184x + 4.9128
R² = 0.9995
y = 0.0141x + 4.5713
R² = 0.9855
RT
LT
Linear (RT)
Linear (LT)

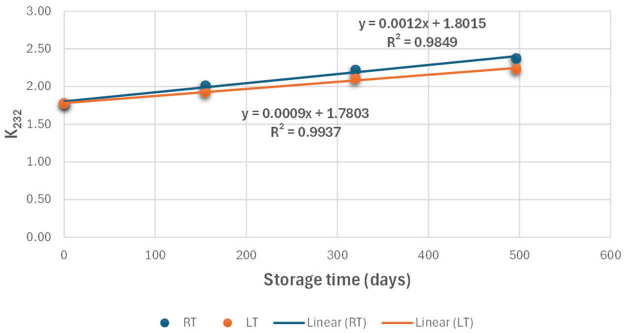
K232
Storage time (days)
y = 0.0012x + 1.8015
R² = 0.9849
y = 0.0009x + 1.7803
R² = 0.9937
RT
LT
Linear (RT)
Linear (LT)

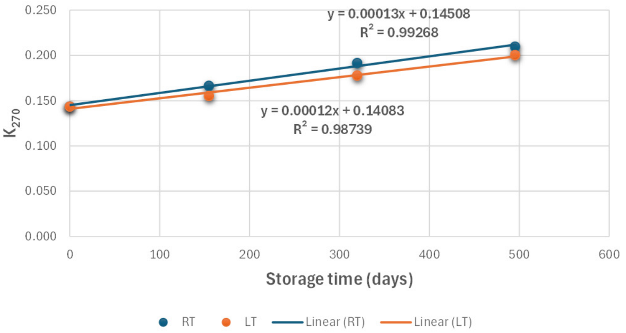
K270
Storage time (days)
y = 0.00013x + 0.14508
R² = 0.99268
y = 0.00012x + 0.14083
R² = 0.98739
RT
LT
Linear (RT)
Linear (LT)

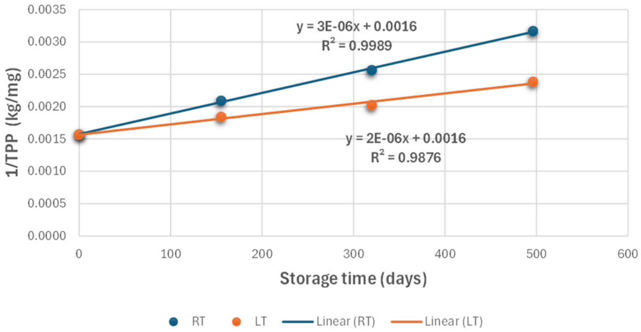
1/TPP (kg/mg)
Storage time (days)
y = 3E-06x + 0.0016
R² = 0.9989
y = 2E-06x + 0.0016
R² = 0.9876
RT
LT
Linear (RT)
Linear (LT)

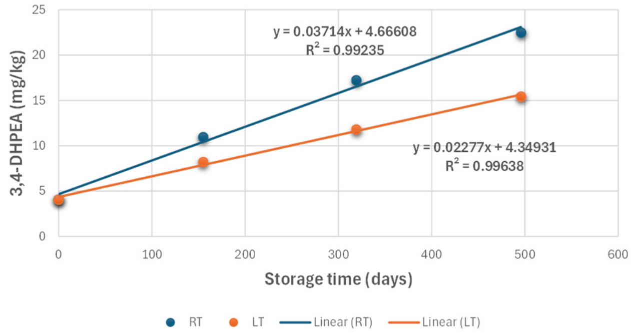
3,4-DHPEA (mg/kg)
Storage time (days)
y = 0.03714x + 4.66608
R² = 0.99235
y = 0.02277x + 4.34931
R² = 0.99638
RT
LT
Linear (RT)
Linear (LT)

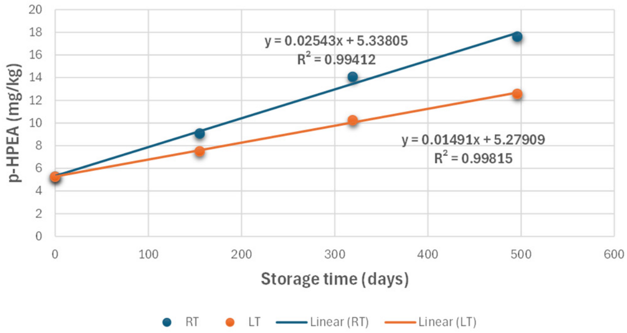
p-HPEA (mg/kg)
Storage time (days)
y = 0.02543x + 5.33805
R² = 0.99412
y = 0.01491x + 5.27909
R² = 0.99815
RT
LT
Linear (RT)
Linear (LT)

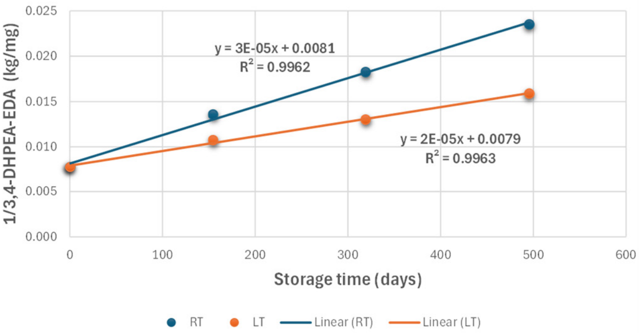
1/3,4-DHPEA-EDA (kg/mg)
Storage time (days)
y = 3E-05x + 0.0081
R² = 0.9962
y = 2E-05x + 0.0079
R² = 0.9963
RT
LT
Linear (RT)
Linear (LT)

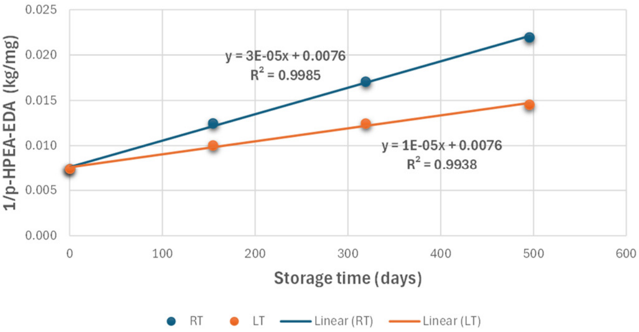
1/p-HPEA-EDA (kg/mg)
Storage time (days)
y = 3E-05x + 0.0076
R² = 0.9985
y = 1E-05x + 0.0076
R² = 0.9938
RT
LT
Linear (RT)
Linear (LT)

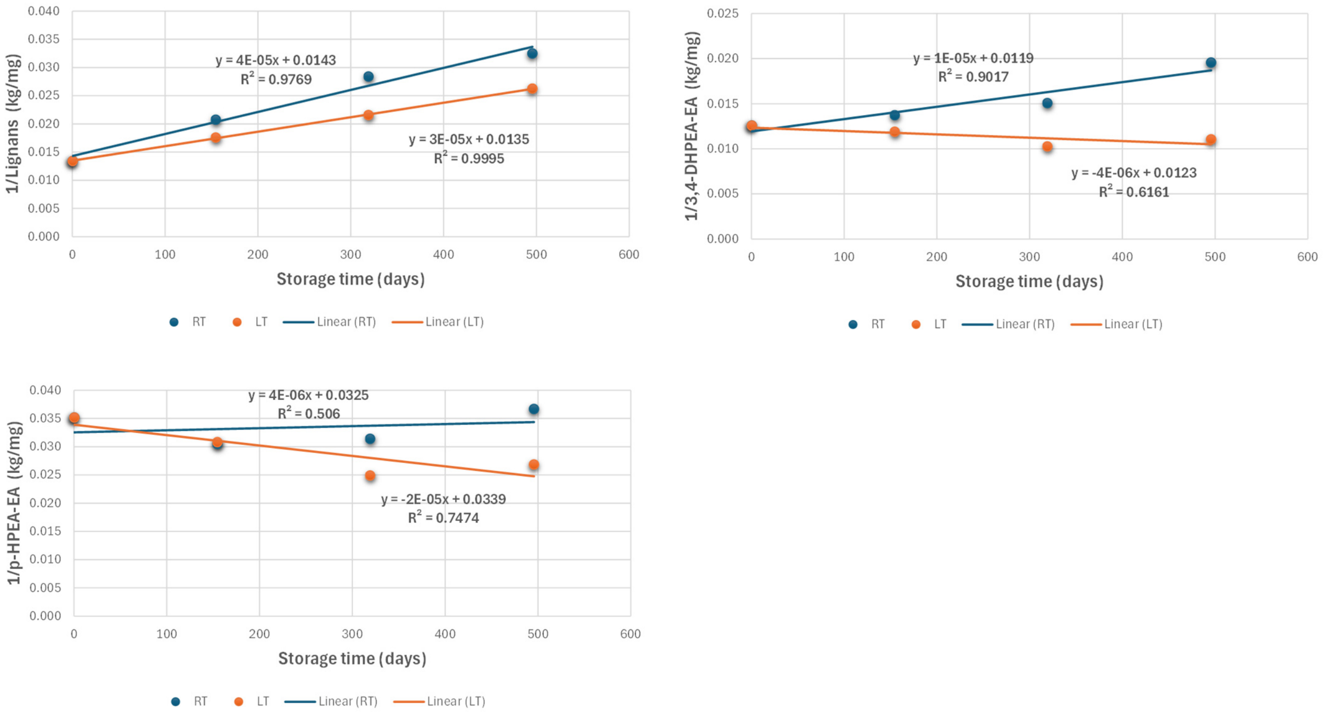

Figure S4. Kinetics of quality and phenolic indicators, averaged along the three years, of Coratina *cv*. EVOO during storage time.
