# Supplementary material for: Effect of Long-Term Storage Temperature on the Quality of Extra-Virgin Olive Oil (Coratina cv.): A Multivariate Discriminant Approach
Source: Antioxidants (Basel). 2025 Nov 19;14(11):1379. doi: 10.3390/antiox14111379 (PMC12649587; doi:10.3390/antiox14111379)
Supplement: Supplementary file 1 [file antioxidants-14-01379-s001.zip › Table S1_Univariate results of full factorial MANOVA.pdf]

Table S1. Univariate results of full factorial two-way MANOVA analysis.

Effect	df	FA		PV		K_{232}		K_{270}		TPP		3,4- DHPEA	
		F	p	F	p	F	p	F	p	F	p	F	p
Storage time	3	0.26	0.8579	378	0.0000	201	0.0000	127	0.0000	130	0.0000	50	0.0000
Storage temperature	1	0.05	0.8168	56	0.0000	28	0.0000	13	0.0004	42	0.0000	19	0.0000
Storage time*Storage temperature	3	0.03	0.9945	7	0.0002	3	0.0176	1.5	0.2108	5	0.0024	3	0.0206

	df	*p*-HPEA		3,4 DHPEA-EDA		*p*-HPEA-EDA		Lignans		3,4- DHPEA-EA		*p*-HPEA-EA	
		F	p	F	p	F	p	F	p	F	p	F	p
Storage time	3	40	0.0000	32	0.0000	55	0.0000	31	0.0000	1.7	0.1668	6	0.0004
Storage temperature	1	15	0.0001	12	0.0007	17	0.0001	9	0.0035	8	0.0070	1.5	0.2194
Storage time*Storage temperature	3	3	0.0391	1.4	0.2441	1.9	0.1279	1.1	0.3663	2	0.0422	0.6	0.062
