# Supplementary material for: Effect of Long-Term Storage Temperature on the Quality of Extra-Virgin Olive Oil (Coratina cv.): A Multivariate Discriminant Approach
Source: Antioxidants (Basel). 2025 Nov 19;14(11):1379. doi: 10.3390/antiox14111379 (PMC12649587; doi:10.3390/antiox14111379)
Supplement: Supplementary file 1 [file antioxidants-14-01379-s001.zip › Table S2_Discriminant function parameters.pdf]

Table S2. Discriminant function parameters to review the independent contributions for each k-variable to the overall discrimination between Coratina EVOOs stored at room temperature (RT) and 4 °C (LT).

	**Wilks' Lambda**	**Partial Lambda**	**F-remove (1, 19)**	**p-level**	**Tolerance**
$\mathbf{k_{2(p\text{-}HPEA\text{-}EDA)}}$	0.4971	0.4932	19.52	0.0003	0.6039
$\mathbf{k_{0(3,4\ DHPEA)}}$	0.4088	0.5998	12.68	0.0021	0.7848
$\mathbf{k_{0(PV)}}$	0.3861	0.6351	10.92	0.0037	0.6060
$\mathbf{k_{2(p\text{-}HPEA\text{-}EA)}}$	0.3462	0.7083	7.82	0.0115	0.5171

The forward stepwise analysis stopped after 4 steps, inserting 4 k-variables in the model.
